# Personality Traits Induce Different Brain Patterns When Processing Social and Valence Information

**DOI:** 10.3389/fpsyg.2021.782754

**Published:** 2022-01-20

**Authors:** Jorge Carlos Hevia-Orozco, Azalea Reyes-Aguilar, Raúl Hernández-Pérez, Leopoldo González-Santos, Erick H. Pasaye, Fernando A. Barrios

**Affiliations:** ^1^Escuela de Psicología, Universidad Anáhuac Mayab, Mérida, Mexico; ^2^Instituto de Neurobiología, Universidad Nacional Autónoma de México, Santiago de Querétaro, Mexico; ^3^Facultad de Psicología, Universidad Nacional Autónoma de México, Mexico City, Mexico; ^4^Department of Ethology, Institute of Biology, Eötvös Loránd University, Budapest, Hungary

**Keywords:** personality, cooperation, representational similarity analysis, empathy, emotional valence

## Abstract

This paper shows the brain correlates of Cloninger’s personality model during the presentation of social scenarios under positive or negative valence situations. Social scenarios were constructed when participants played the Dictator game with two confederates that had two opposites roles as the cooperator (Coop) and non-cooperator (NoCoop). Later the same day during a fMRI scanning session, participants read negative (Neg) and positive (Pos) situations that happened to confederates in the past. Participants were asked to think “how do you think those people felt during that situation?” A dissimilarity matrix between stimuli were obtained from fMRI results. Results shown that Harm Avoidance trait people make use of right middle frontal gyrus and left superior frontal gyrus to discriminate between Coop and NoCoop. Cooperation as a trait makes use of the right superior temporal gyrus and the right precuneus to discriminate between Coop and NoCoop in positive social scenarios. Finally, Self-directedness trait people make use of the right inferior parietal lobe to discriminate between Coop and NoCoop in negative social scenarios and the right precuneus to discriminate between Coop and Strangers. An intuitive link between discrimination findings and behavioral patterns of those personality traits is proposed.

## Introduction

Personality traits refer to the individual differences in behaving, feeling and thinking in certain consistent ways. They represent behaviors that that allow us to successfully or unsuccessfully adapt to diverse situations (Buss, 1996). It is highly important to peruse how different personality traits lead to differences in perceiving and processing social information, mainly through the lens of empathy. Moreover, personality has a massive impact on criminal behavior (Van Gendel and De Vries, 2012), psychiatric disorders like depression (Cam Celikel et al., 2010), risky decision making (Lauriola et al., 2013), coping with stress (Fauerbach et al., 2005), adaptability to social environments (Buss, 1996), etc. Therefore, more research is needed for clarifying important aspects of this topic, for example, how the brain discriminates social stimuli in participants with different personality traits.

One of the main accepted models for personality is the one that was postulated by Cloninger (1986). This model includes dimensions like Novelty Seeking (NS), Harm Avoidance (HA), and Reward Dependence (RD). This tripartite—personality model was later expanded to include the RD subscale, Persistence (PE) with features independent from the former (De Fruyt et al., 2000). Self-directedness (SD), Cooperativeness (CO), and Self-transcendence (ST) were finally included to explore character dimensions. More details about this model can be found elsewhere (Kose, 2003). Since personality is an individual mediator of our reactions to information from the complex environment, it is necessary to enrich our knowledge about how different personalities encode highly salient information like social and valence information.

Regarding brain functional imaging that sustain personality traits there are few studies that have specified those areas that are important for the Cloninger personality model. Talking about temperament traits, the HA trait has been negatively and positively related with the amygdala while watching negative pictures (Baeken et al., 2009, 2010), and while paying attention to tasks with emotional distractors (Most et al., 2006); the NS trait has been positively related with the hippocampus when participants were watching familiar stimuli and expected rewards (Krebs et al., 2009) but also whit novel stimuli (Naghavi et al., 2009). Also, RD trait has been positively related with the ventral striatum and the orbitofrontal cortex when participants were anticipating a big monetary reward (Hahn et al., 2009). In relation with character traits, SD participants have been negatively related with the amygdala when processing pictures of food versus scrambled images (Grimm et al., 2012) and regarding Persistance, a higher activity was found in the orbitomedial prefrontal cortex and ventral striatum when participants were watching negative stimuli (Gusnard et al., 2003). The Transcendence trait, a poorly defined construct, has been related with the dorsal attentional system and with the hippocampal- cortical memory system (Vago and Silbersweig, 2012). Finally, although through anatomical standards, the Cooperativeness trait has been related with connectivity between caudate and anterior cingulate cortex and ventrolateral prefrontal cortex (Lei et al., 2016). Those findings manifest that, distinct personalities have different patterns of brain activity however, neither of those functional studies describe how brain discriminate between stimuli, which would be very helpful to delving about our adaptability to real world scenarios. This is important since personality traits are crucial for people for handling social and complex scenarios and might influence the appearance of psychiatric conditions (Mizuno-Matsumoto et al., 2012).

Remember the last time when you watched a Hollywood movie. In the one hand, what did you feel when the meanest villain ever was killed by the main superhero of the movie? Pleasure, right? But in the other hand, what did you feel when the superhero was killed by the villain of the movie? Sorrow, anger, right? We evoke similar feelings in real life when we know in the one hand about a serial killer that was killed or when in the other hand a policeman is murdered. A basic process that we need to evoke those feelings is the ability to discriminate or to distinguish between those pair stimuli: cooperators or no cooperators and positive or negative situations and distinct personality traits might use different brain regions to encode that discrimination ability.

A technique that permits us to distinguish brain activity that carry this type of opposite stimuli is the representational similarity analysis or RSA. With this tool, we can reveal how brain areas represent information by comparing the correlational distance between a pair of stimuli in a confusion matrix (Popal et al., 2019). In the context of social information and beyond the discrimination of low-level physical characteristics as body parts, previous work has been done to reveal how certain brain areas differentiate between socially abstract concepts, like affect properties of visual scenes (Chikazoe et al., 2014), shearing emotional mental states (Nummenmaa et al., 2012), gender and race (Stolier and Freeman, 2016), socio—moral strategies (Van Baar et al., 2019).

The innovation and the relevance of this research project is the advance in the knowledge of the discrimination mechanism that each of us make. We are constantly establishing differences between stimuli, and this is because our need of adaptation. Hanging out with non-cooperative people and to repeal cooperative people will not be a good decision in several ways. This decision begins with the ability to discriminate both types of stimuli, however, we all have different personalities and as we have mentioned before, distinct personalities processes stimuli in different manners. In a clinical sense, this is also important because of the need of psychiatric patients to discriminate between relevant stimuli as in depression or anxiety patients (Mizuno-Matsumoto et al., 2012). Their capacity to discriminate between cooperation and non-cooperation and between positive and negative situations will give them more chances for their adaptability.

However, the way we make the discrimination between social and valence stimuli could be based on the way we are, this is, is based on who we are, in other words, in our personality. Therefore, the objective of this study was to reveal if different personality traits can discriminate between conceptually opposites social contexts (cooperators and no cooperators) that are immersed in opposites positive vs. negative situations. We hypothesized that different RSA patterns regarding to social (cooperation vs. no cooperation) and valence stimuli (positive vs. negative), will be revealed according to different personality traits.

## Materials and Methods

### Participants

Participants in this study were 34 volunteers between 25 and 35 years old (17 women) (*M* = 28.94, SD = 3.00), and all were right-handed (Edinburgh Handedness Inventory, Oldfield, 1971, *M* = 42.56, SD = 11.34) and native-Spanish speakers, participated in this study. No neurological or psychiatric disorders were detected using the Mexican version of the Symptom Check List 90 (González-Santos et al., 2007). Before starting the experiment, participants signed up the informed consent in accordance with the local ethics committee on the Use of Humans as Experimental Subjects (Table 1). The participant sample size was calculated using the “power.f2.test” function as part of the “pwr” package in R, it was with degrees of freedom for numerator (u) = 4, effect size (f2) = 0.6 (medium), sig.level = 0.01, and power = 0.8 high, for general linear model. The result was v = 29, which is *n* = 4 + 29 + 1. Sample size = 34.

**TABLE 1:** Participant’s demographic information.

Demographic information	Frequencies
**Gender**	
Female	17
Male	17
**Occupation**	
Student	23
Worker	11
**Marital status**	
Married	7
Single	21
Divorced	1
Free union	5
**Last degree of studies**	
Bachelor’s degree	13
Master’s degree	19
Doctorate	2

### Behavioral Task

As soon as participants arrived at the laboratory, a picture of them with neutral face was taken. The argument for this step is that it would conform a pool of participant’s pictures for future. Also, participants give a group of 10 positive, negative and neutral situations that happened in the previous 6 months for another pool of emotionally valence situations that would be used in future investigations. Then, participants played the iterated version of the Dictator Game (DG) with two other participants (confederates) in our laboratory. Without the knowledge of the participant, two different roles were assigned by the experimenter to the confederates: one of them had the role of cooperator and the other the role of non-cooperator. The game was played two times (back-to-back) and on each round the role of dictator was rotated among the three players. For the Dictator Game a money component is needed, therefore, players completed a task before each trial (i.e., make a paper case for CDs), and received a payment, the same amount for each player ($4 MXN). On each trial, the appointed dictator determined the redistribution of the payment of all players, i.e., dictator could give money from her/him own payment to other players (cooperative strategy) or could take money from payment of others two players to herself-himself (non-cooperative strategy). In each round, one confederate was instructed to use a cooperative strategy while the other was instructed to use a non-cooperative strategy for re-distribution of payment. With this method, experimental participants would acquire two opposites attributes from the confederates: cooperation and non-cooperation. More features and the precise setup have been described elsewhere (Reyes-Aguilar et al., 2017). To later evaluate the participant response to the different confederate situations in the scanning task, a picture was taken of the participant at the end of the Dictator Game and a picture of the confederates was taken the beginning of the project (all with neutral facial expressions). With this strategy, scanner task would be more credible since they were instructed that positive and negative situations had happened to previous participants. During scanner task, participants realized that among previous participants the confederates were let the participants know that confederated had participated on this study in previous days.

### Scanning Task

Details of the scanning task have been described elsewhere (Reyes-Aguilar et al., 2017). In brief, a 2 (confederate: Coop vs. NoCoop) × 2 (situations: positive vs. negative) was constructed for the scanning task. For the positive and negative hypothetical scenarios, emotional valence situations were previously constructed (Reyes-Aguilar and Barrios, 2016) and 62 positives, 62 negatives and 49 neutrals were used for the initial project (neutral scenarios were no included on this second analysis). This design resulted in four experimental conditions: (1) the cooperative confederate in positive situations (CPos) and (2) cooperative confederate in negative situations (CNeg), and (3) the non-cooperative confederate in positive situations (NCPos) and (4) the non-cooperative confederate in negative situations (NCNeg). During this activity, participants read descriptions of negative or positive situations and then watched pictures of faces, including the confederate’s pictures (taken before project beginning) while imaging at the same time “how should that person feel in the situation that you just read.”

Four runs were tested and each of them contained 68 events of which five events were for each experimental condition combined with 48 control background events, and lasted 9.5 min. An experimental event trial within the fMRI scanning session consisted of an emotional situation followed by a picture of a confederate or stranger and has been presented elsewhere (Reyes-Aguilar et al., 2017).

Regarding following instructions in the scanner task, there are different ways to approach to this issue. Before beginning that scanning task, participants were instructed to press a button indicating that they have just read the sentences given on each trial and describing each social situation. Although, these instructions were given before scanning, a fluid communication between bloc stimuli was present between experimenter and participants through the inside – outside communication system of the scanner. This allow researchers to remember constantly the indications for task execution to participants and to questioning if they were correctly answering the task.

Also, as reported by Reyes-Aguilar et al. (2017), brain activity during each trial was presented in a brain network that has been broadly related with mentalization tasks. This was expected according to the instruction given to participants: think about “how that person should feel in the situation that they just read.” Therefore, is natural to think that brain network activity reflected the instructions given during the task. Also, after scanner tasks, participants responded a likert scale where they indicated the intensity of their liking for the two confederates on a 5-point Likert scale, ranging from 1 (no liking) to 5 (high liking).

### Image Acquisition and Data Preprocessing

fMRI imaging was performed on a 3.0T GE MR750 instrument (General Electric, Waukesha, WI, United States) using a 32-channel head coil. High-resolution structural 3D-T1-weighted images were acquired for anatomical localization covering the whole brain. Data processing was performed with FMRI Expert Analysis Tool using FMRIB’s Improved Linear Model (FEAT FILM) Version 5.98 and each participant’s data were motion and slice timing corrected and normalized onto MNI common brain space (Montreal Neurological. More details about data processing are detailed in Reyes-Aguilar et al. (2017).

### Cloninger Temperament and Character Inventory

The personality test was presented via online and participants answered according to self-report questions. Cloninger’s Psychobiological Model of Temperament and Character (Cloninger, 1986), was first developed to provide a multiple—level model that provide a strong background for personality regarding genetics, neurobiology and cognitive structures. This is a binomial (“Accept” or “Reject”) 240—item questionnaire that reveals facets of 3 aspects of temperament, Harm Avoidance (HA), Novelty Seeking (NS) and Reward Dependence (RD), and 4 aspects of character traits, Self-directedness (SD), Cooperativeness (CO) and Self-transcendence (ST). Items from this test were classified and used as the independent variable.

### Representational Similarity Analysis

To calculate the representational similarity between stimulus pairs, we created dissimilarity maps for each participant by using a searchlight approach (Kriegeskorte et al., 2008) and a spherical kernel. For a given participant, on each voxel, we created a sphere (*r* = 4 voxels), using the activity pattern of the voxels within the sphere for two categories across runs. As a measure of dissimilarity for that voxel, we calculated the correlation distance (1 – Pearson correlation). The dissimilarity was projected onto a different map in the coordinates representing the center of the sphere. This procedure was repeated for every voxel within the brain, thus creating a dissimilarity map, and then repeated for each participant, creating a dissimilarity map for each participant and each stimulus pair.

To assess the relationship between the dissimilarity of a stimulus pair and the behavioral scores, we created a correlation map for each of the temperament and character dimensions. On a given voxel, we calculated the correlation between the dissimilarity of a stimulus pair (Pos, Neg, Coop, NoCoop, Str) and the temperament and character dimensions across participants and projected the resulting correlation onto a different map; we repeated this procedure for each voxel, thus creating a correlation map. The resulting map then represented the correlation between the temperament and character dimensions and the representational similarity of a pair of stimulus categories.

Given the number of correlations, some spurious correlations were expected, so to assess the number of correlations expected by chance, we used a permutation-based approach similar to one described previously (Stelzer et al., 2013). That is, we calculated the probability of obtaining a given correlation score under a no-signal condition. We calculated random correlation maps by repeating the same procedure described above but randomly swapping the stimulus labels on each participant prior to calculating the dissimilarity maps. We repeated this procedure 1,000,000 times, thus creating a random correlation map for each repetition. We then compared the correlation found for each voxel with the distribution of correlations in the random correlation maps. The values for each voxel were then converted to Z-scores and threshold at *p* < 0.001. Using the random correlation maps and the same threshold, we calculated the cluster size distribution under a no-signal condition, and then used this distribution to assess the probability of each cluster size found; we then thresholded the maps using a cluster size *p* < 0.05.

## Results

### Behavioral Results

In the context of the 3 temperament traits, regarding Novelty Seeking (NS) one male and two females scored below normal standards for Mexican population and one men above standard. Regarding Harm Avoidance (HA) four males scored above the norm and for Reward Dependence (RD) eleven males and eight females pointed above the norm. Regarding the 4 character aspect, in Persistence (PS) one male pointed above the norm and three males and five females scored below the norm, related with Self-Directedness (SD) scores, eight males and five females pointed below the norm; for Cooperativeness (CO) two males and one female scored below the norm and for Self-Transcendence (ST) trait, 2 males and 3 females scored below the norm and 2 males and 2 females scored above the norm.

### RSA Results

**FIGURE 1:**
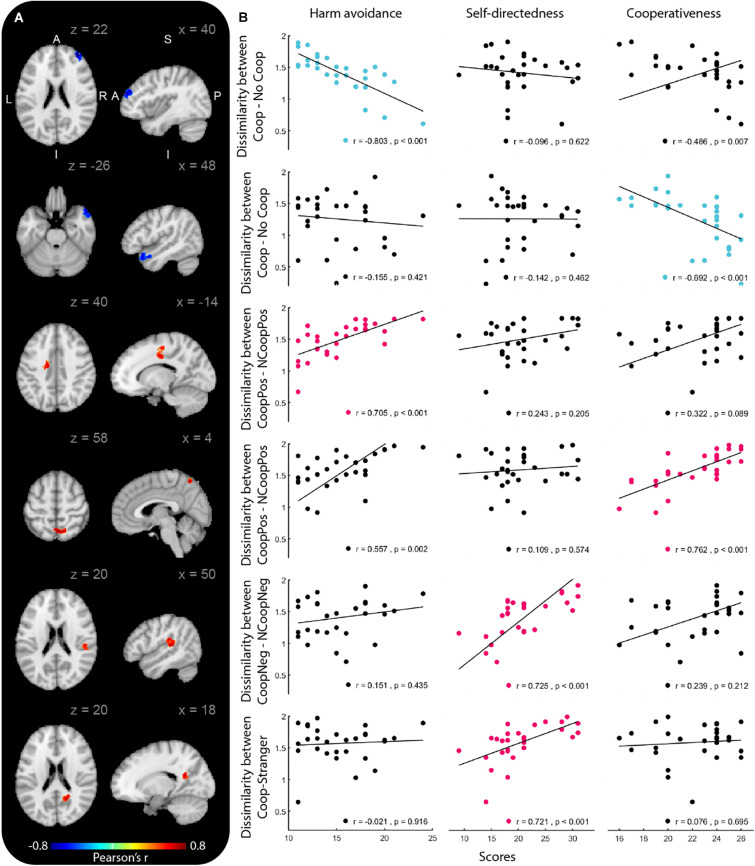
Brain regions whose dissimilarity between stimuli pairs correlated with temperament or character dimensions. Group-level correlation maps (*n* = 29) and relationship between dissimilarities and temperament dimensions (Harm avoidance, Self-directedness and Cooperativeness). Only clusters that showed significant correlation (permutation test, *p* < 0.001; cluster corrected at *p* < 0.05) with the temperament dimensions are shown. The results are overlaid in the MNI-152 atlas.

We identified the brain regions that showed a relationship between the temperament and character dimensions and the representational similarity between stimulus pairs (Figure 1). When testing the dissimilarity between Coop and NCoop, we found two clusters, both with significant negative correlations. The first cluster correlated with the HA dimension and its peak was located in the right middle frontal gyrus (MFG), while the second cluster correlated with the CO dimension and its peak was found in the right superior temporal gyrus (STG). No other correlations were found for the remaining temperament dimensions.

When testing the dissimilarity between CoopPos and NoCoopPos, we found two clusters with positive correlations. The first cluster correlated with the HA dimension and its peak was located in the left superior frontal gyrus (SFG); the second cluster correlated with the CO dimension and its peak was found in the right precuneus (PCun). When testing the remaining temperament dimensions, no other significant clusters were found.

When testing the remaining dissimilarity pairs, we found two clusters that correlated with the SD dimension, both positively. The first cluster was related to the CoopNeg and NCoopNeg dissimilarity with a peak in the right inferior parietal lobe (IPL). The second was related to the dissimilarity between the Coop and the stranger and its peak was found in the right PCun. No other stimuli pairs nor temperament dimensions revealed significant clusters (Table 2).

**TABLE 2:** Brain regions whose dissimilarity between stimuli pairs correlated with temperament or character dimensions.

Comparison	Variable	Brain region	Cluster size	Pearson correlation	Coordinates (x, y, z)
Coop-NCoop	HA	MFG	186	–0.803	40, 54, 22
Coop-NCoop	CO	STG	215	–0.692	48, 20, –26
CoopPos-NCoopPos	HA	SFG	163	0.705	–14, –12, 40
CoopPos-NCoopPos	CO	PCun	185	0.762	4, –60, 58
CoopNeg-NCoopNeg	SD	ROL	158	0.725	50, –26, 20
Coop-Stranger	SD	PCun	159	0.721	18, –52, 20

*Threshold for reporting a cluster as significant is p < 0.001 (permutation test, n = 10,000), cluster corrected at p < 0.05. L, left; R, right; MFG, Middle frontal gyrus; STG, Superior temporal gyrus; SFG, Superior frontal gyrus dorsolateral; PCun, Precuneus; ROL, Rolandic operculum.*

## Discussion

In this study we found different personality patterns among a group of young adults. Only a qualitative approach was used for analyzing the behavioral data. Through this perspective, we got different personality patters that were above and below the norm for Mexican population. Our main analysis was based on RSA findings associated with personality traits. We used neuroimaging data to explore how different personality scores are correlated with specific patterns of neural discrimination between cooperation and non-cooperation. We found that three personality traits have significant correlation: the harm avoidance with medial frontal cortex and the superior frontal cortex; the cooperativeness with the superior temporal gyrus and with the precuneus and the self-directedness with the Rolandic operculum and with the precuneus.

Among executive functions, inhibitory control has a pivotal role in the HA trait. Izadpanah et al. (2016) demonstrated that inhibition was the mediator between HA scores during adolescence and the development of emotion regulation strategies during early adulthood. Also, Wierenga et al. (2014) showed a lower activation of rMFG and more inhibitory errors during hard trials in patients with anorexia nervosa (a high HA trait participants) compared with adolescents from a group comparison, when participants performed an emotional Go/No-Go task. Those findings highlight not only the relevance of inhibition for the HA trait, but also the participation of rMFG in normal inhibitory abilities. Other studies have related rMFG with inhibitory abilities (Nakata et al., 2008; Berkman et al., 2009). Nevertheless, what is the importance of inhibitory control for the discrimination between cooperators and no cooperators? People with high levels of HA have been described as cautious when forming social relationships with the objective of reducing the risk of being humiliated (Cloninger, 1986). Under this behavioral context, the inhibitory control would cause people to avoid certain actions that would put them in awkward or embarrassing conditions. Therefore, the lower discrimination between cooperators and no cooperators in higher HA trait might be due to the reduced contribution of inhibitory control, manifested in our study by the pattern of representation in the rMFG. With this idea, we could consider that social situations are intrinsically challenging conditions for high HA scorers, so they cannot discriminate between cooperators and no cooperators with neural mechanisms related to inhibitory control. Therefore, lower social adaptability of HA participants might be related with the fact that they cannot easily make the discrimination between cooperators and no cooperators or, in other words, friends or foes with neural mechanisms related to inhibitory control.

However, there might be some situations that would allow high HA scorers to feel more attuned toward making a social discrimination, for example, during positive social interactions. As in our positive context stimuli, Ashby et al. (1999), made the proposal that positive affect stimuli improve performance in several cognitive tasks. For example, cognitive flexibility has been shown to be enhanced when participants were previously exposed to pleasant pictures like lovely babies or beautiful scenes, compared to when participants were exposed to negative or neutral pictures (Wang et al., 2017). Also, positive affective stimuli have an enhancement effect on creative problem solving (Isen et al., 1987), integration of information (Estrada et al., 1997), attentional selection (Rowe et al., 2007), etc. To explain this phenomenon, Ashby et al. (1999) proposed the dopaminergic theory of positive affect. They postulated that during this condition, dopamine is released from the ventral tegmental area to prefrontal regions and that this effect would facilitate the executive function performance. This proposal coincides with the findings of Fehr et al. (2014), who found an activation of the lSFG when participants were watching a social—positive videoclip. Taking together these arguments we can assume that the pattern of representation of the lSFG is reflecting a positive tuning state for high HA scorers to make the discrimination between cooperators and no cooperators. In terms of social adaptability, perhaps HA participants are taking advantage of positive scenarios for improving their capacity of discrimination so they can feel safer in taking the next step toward pursuing their goals.

CO participants used the right superior temporal gyrus (rSTG) for discrimination between cooperators and no cooperators and precuneus for the discrimination between cooperation and no cooperation in positive context. The CO behavioral pattern relies on the identification with and the acceptance of other people. We can speculate that for the sensibility to identify people with different behavioral features, highly sharpened skills are needed for CO participants to semantically classify people in different categories. This speculation is in line with Suzuki et al. (2011). They found that the temporal pole is activated when participants cooperate with other cooperators or even with strangers but not with non-cooperators. They speculate that this area is closely related with making the discrimination between behavioral features. Moreover, previously reported activity of the anterior superior temporal gyrus is in line with this explanation. It has been broadly related not only with semantically classification of abstract concepts (Jefferies et al., 2012), but mainly in social information perception (Zahn et al., 2007; Wilson-Mendenhall et al., 2013), like lack of empathy (Seehausen et al., 2014), trust (Schjoedt et al., 2011), and cooperation (Lissek et al., 2008). Perhaps the lower discrimination between cooperators and no cooperators put CO participants in the right place to be empathic, helpful and supportive with both types of people (Kose, 2003). That is, since CO participants behavioral profile is highly empathic and sympathetic, perhaps CO participants conceive both cooperators and no cooperators as no different between each other so they can be subjects of their cooperative behavior.

However, CO participants make use of the precuneus for making the discrimination between cooperators and non-cooperators but in positive contexts. CO participants are also considered as tolerant to the opinions of others and those who treat people with dignity, respect and compassion. The core function for this behavior is the theory of mind (ToM), i.e., the capacity to infer the mental states of others (Schurz et al., 2014). The participation of the precuneus is expected according to its strong involvement in inferring the mental state of others, as when deception is perceived during a cooperation task (Lissek et al., 2008). Inferring other’s intentions is imperative for discrimination between cooperation and no cooperation for our social adaptation. However, what is the particularity of a positive stimulus for CO participants for making the discrimination between cooperators and no cooperators? One possibility is the functional connectivity that precuneus has with the amygdala (Kumar et al., 2014), that has been demonstrated that also manifest a higher activation when a positive stimulation is presented (Bonnet et al., 2015) and this might be producing a pivotal role of the precuneus when participants are processing social and positive information. More studies are needed to reveal the participation of this connectivity under the context of personality. This will be helpful to shed light on the relationship between personality and psychiatric illnesses in socially relevant contexts, since in some psychiatric illnesses it is important to know how positive stimuli bias social evaluation in some patients.

Self-directedness as personality trait describes features of individual behavior but not social behavior as cooperation or harm avoidance traits do (Garcia et al., 2017). However, what are the implications of the rIPL of participants with self-directedness personality trait for discriminate between cooperators and non-cooperators in negative contexts? First, self-directedness is defined as the ability to develop good habits and behave in accordance with long-term values and goals. This personality trait evaluates levels of goal-directed behavior and delay in gratification in decision—making. This means that those participants constrain their thoughts to them self and their goals, in opposition to external influences (Kennis et al., 2013). In a social interaction it is plausible that self-directedness trait make use the self as a referential starting point for making inferences about others; this is for understanding others mental state, these participants are first using a self-reference for making the distinction between the self and others (van der Cruijsen et al., 2017). This is a typical function of the mirror neuron system (MNS) and the cortical midline structures (CMN). According to Uddin et al. (2007), the MNS provides a mapping for differences between the self and the other in a physical aspect and the CMN maintain, and support processes that simulate other’s complex psychological aspects, such as attitudes, perhaps by simulation of one’s own attitudes. Interestingly our other significant area for self–directedness trait is an area considered into the CMN like the precuneus, for the discrimination between cooperators and strangers. This will mean that our findings in self-directedness are revealing the implications of two hubs of the MNS and the CMN, like the rIPL and the right precuneus, respectively.

Our findings go in the same line as what Trapp et al. (2014) found. They explore the involvement of three entities in one interaction: self, other and an object (what they called the tripartite engagement). They found the participation of the IPL when participants were thinking about how and object can be used in a social interaction with another person and the precuneus when participants were thinking about how an object can be used by others. In our case, the rIPL can be encoding the difference between cooperator and non-cooperators taking the self as a reference (as in a tripartite engagement) and the precuneus for encoding the mental state of the cooperator compared with a stranger (as a process for recognize the mental state of other).

## Data Availability Statement

The raw data supporting the conclusions of this article will be made available by the authors, without undue reservation.

## Ethics Statement

The studies involving human participants were reviewed and approved by the Bioethics Committee of the Neurobiology Institute, UNAM. The patients/participants provided their written informed consent to participate in this study.

## Author Contributions

JH-O, AR-A, and FB developed the study concept and contributed to the study design. JH-O and EP ran the experiment. LG-S and EP performed data administration and MR work. JH-O, AR-A, and RH-P performed data analysis and interpretation. JH-O and AR-A wrote the manuscript draft. RH-P prepared the figure. AR-A ran the experiment. All authors provided critical revisions and approved the final version of the manuscript.

## Conflict of Interest

The authors declare that the research was conducted in the absence of any commercial or financial relationships that could be construed as a potential conflict of interest.

## Publisher’s Note

All claims expressed in this article are solely those of the authors and do not necessarily represent those of their affiliated organizations, or those of the publisher, the editors and the reviewers. Any product that may be evaluated in this article, or claim that may be made by its manufacturer, is not guaranteed or endorsed by the publisher.
